# A compact high-resolution X-ray powder diffractometer

**DOI:** 10.1107/S0021889813027313

**Published:** 2013-11-15

**Authors:** Paul F. Fewster, David R. D. Trout

**Affiliations:** a PANalytical Research Centre, Sussex Innovation Centre, Falmer, Brighton, East Sussex BN1 9SB, UK

**Keywords:** high-resolution powder diffaction, transmission geometry, monochromatization

## Abstract

A new powder diffractometer operating in transmission mode is described. It can work as a rapid very compact instrument or as a high-resolution instrument, and the sample preparation is simplified. The incident beam optics create pure Cu *K*α_1_ radiation, giving rise to peak widths of ∼0.1° in 2θ in compact form with a sample-to-detector minimum radius of 55 mm, reducing to peak widths of <0.05° in high-resolution mode by increasing the detector radius to 240 mm. The resolution of the diffractometer is shown to be governed by a complex mixture of angular divergence, sample size, diffraction effects and the dimensions of the detector pixels. The data can be collected instantaneously, which combined with trivial sample preparation and no sample alignment, makes it a suitable method for very rapid phase identification. As the detector is moved further from the sample, the angular step from the pixel dimension is reduced and the resolution improves significantly for very detailed studies, including structure determination and analysis of the microstructure. The advantage of this geometry is that the resolution of the diffractometer can be calculated precisely and the instrumental artefacts can be analysed easily without a sample present. The performance is demonstrated with LaB_6_ and paracetamol, and a critical appraisal of the uncertainties in the measurements is presented. The instantaneous data collection offers possibilities in dynamic experiments.

## Abstract 2

A new powder diffractometer operating in transmission mode is described. It can work as a rapid very compact instrument or as a high-resolution instrument, and the sample preparation is simplified.

## 1. Introduction

High-resolution X-ray powder diffractometry enables closely spaced peaks in an X-ray diffraction pattern to be isolated, allowing greater certainty in the identification of phases present in powdered material. The purpose of high-angular-resolution methods is to reduce the width of the diffraction lines, which has particular relevance for samples containing a combination of phases with closely spaced peaks, arising from similar crystal plane spacings. High resolution is also relevant for studying powders with large crystal lattice parameters that have many peaks and low-symmetry space groups, *e.g.* triclinic and monoclinic. The peaks in a powder diffractogram are broadened from several contributions, namely, sample-related aspects such as crystallite size, strain effects, instrumental effects associated with its geometry and wavelength dispersion. Initially, the geometries of typical current configurations are discussed, followed by a description of complications of wavelength dispersion and how this is tackled. Finally, the merits of the proposed compact high-resolution X-ray diffractometer are presented.

### 1.1. Current methods in powder diffraction

**Figure 1:**
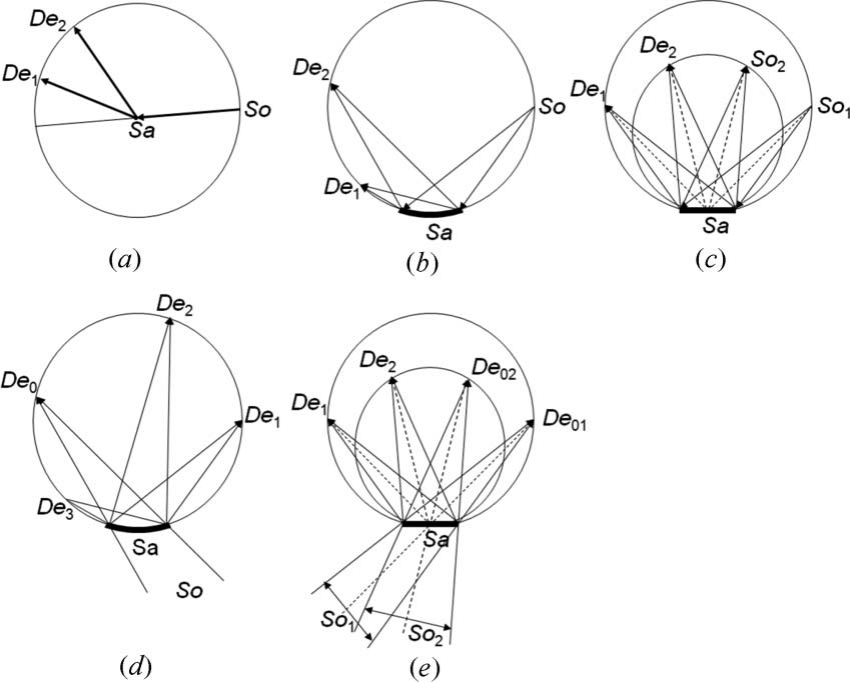
The various geometries for powder diffraction, where *S*_o_ represents the source or a restricted incident beam slit: (*a*) Debye–Scherrer, (*b*) Seemann–Bohlin, (*c*) Bragg–Brentano, (*d*) transmission Seemann–Bohlin and (*e*) transmission Bragg–Brentano.

The discovery of X-ray scattering from fine powders was pioneered by Debye & Scherrer (1916 ▶) and the simplest geometry is generally the layout of the Debye–Scherrer camera. This camera operates by placing a small sample in the centre of a cylinder of film (or a position-sensitive detector) (see Fig. 1 ▶ *a*). The resolution can be increased by careful collimation of the incident beam and improving the ratio of the sample diameter to the detector radius. The sample dimensions ideally should be small since, as the radius is increased, the path length is increased, with a consequent loss in collected intensity. Similarly, the intensity diminishes with the degree of collimation, since longer slit separations are necessary. This geometry, in its simplest form, is unsuitable for high-resolution data collection, because the sample-to-detector distance needs to be large and the sample to be small. In practice, the sample is usually mounted in a capillary or on the outside of a glass fibre, resulting in typical sample sizes of 350–700 µm diameter. Therefore, to achieve peak widths less than 0.1° would require radii of >200 or >400 mm, respectively, provided that the incident beam has no divergence and there is no wavelength dispersion and no microstructure broadening.

The favoured method for achieving high-resolution powder diffractometry requires focusing geometry; this helps to maintain intensity and can more easily include some degree of monochromatization. To achieve the focusing condition, the sample, the divergent point of the incident beam and the convergent point of the scattered beam should lie on the circumference of a focusing circle (Fig. 1 ▶ *b*). This is the principle of the Seemann–Bohlin parafocusing geometry (Seemann, 1919 ▶; Bohlin, 1920 ▶). This clearly allows an incident beam from the source placed on the focusing circle, or entering through a narrow slit, at *S*_o_, to scatter from a curved sample and come to a focus on the circle at positions *D*_e1_, *D*_e2_ *etc*. The beam paths for each position *D*_e_ are scattered through the same scattering angle (2θ). This configuration requires a sample bent to the radius of the circle or one that is very small in comparison with the radius of the focusing circle. The path length and quality of focusing can be difficult to maintain in practice, however, it does allow parallel data collection by placing film or position-sensitive counter detectors around the focusing circle. If the sample is flat this focusing condition is not precise enough to achieve high resolution, unless the instrument has very large path lengths.

To overcome the problem of having a flat sample, the incident and scattered beams can be kept symmetrically related, so that the incident angle onto the sample is half the scattering angle 2θ and the focusing condition is maintained (Bragg, 1921 ▶; Brentano, 1946 ▶). This is the basis of the Bragg–Brentano arrangement. However, to capture peaks at differing 2θ values does require rotation of the sample and the detector and, therefore, the data cannot be collected in parallel (Fig. 1 ▶ *c*). This setup is suitable for large samples. This geometry becomes problematic at low angles without heavily restricting the incident beam divergence, although this can be done automatically with variable slits linked to the incident angle, effectively maintaining the same area on the sample visible to the incident beam.

Both these latter methods, the Seemann–Bohlin and the Bragg–Brentano, use reflection geometry, which can be a problem for some low-absorbing materials in that the penetration will effectively move the sample off the focusing circle and reduce the resolution. Also the resolution depends strongly upon the focus size and the receiving slit dimension, *i.e*. the finite size of *S*_o_ and *D*_e_ in Figs. 1 ▶(*b*) and 1 ▶(*c*). For a typical diffractometer with a radius of 240 mm and a receiving slit of 0.25 mm, negligible focus size, and no wavelength dispersion, a resolution of 0.1° can be achieved.

To recover the high resolution, which is lost to the apparent sample displacement in low-absorbing samples, it is possible to use a convergent incident beam in transmission mode, where again the sample and detector lie on the focusing circle (Fig. 1 ▶ *d*) (Guinier, 1937 ▶, 1952 ▶). This is analogous to the Seemann–Bohlin geometry (Fig. 1 ▶ *b*), but in transmission mode. The Bragg–Brentano transmission version in Fig. 1 ▶(*c*) is given in Fig. 1 ▶(*e*), *i.e.* for a flat sample and, as before, the focus is maintained by scanning and maintaining the symmetrical relationship (Hoppe, 1947 ▶).

### 1.2. Removing wavelength dispersion in current methods

All the geometries above suffer from significant broadening owing to the wavelength spread. To remove some of this wavelength dispersion, *e.g.* isolating the *K*α_1_ component of the *K*α_1_–*K*α_2_ doublet, requires some level of monochromation. Guinier (1937 ▶, 1952 ▶) added a curved single crystal to the Seemann–Bohlin camera to isolate the *K*α_1_ component, and the beam from this was brought to a focus at *S*_o_ in Fig. 1 ▶(*b*). This gave a very useful moderate-to-high-resolution camera. Siddons *et al.* (2008 ▶) have used this geometry in combination with a multilayer mirror to achieve high-resolution powder diffractometry on a synchrotron, with the advantage of parallel detection using solid-state Si-strip detectors on the focusing circle. The resolution is enhanced considerably with the larger beam paths possible, compared with that achievable in a laboratory.

To improve the wavelength dispersion in the Bragg–Brentano geometry of Fig. 1 ▶(*c*), the convergent focusing can be achieved with a bent single crystal as in the Guinier camera (Johannsson, 1933 ▶). Since the intrinsic diffraction width of a single crystal is typically 0.003°, the *K*α_1_ component of the *K*α_1_–*K*α_2_ doublet can easily be isolated and focused onto the incident beam slit at *S*_o_ (Fig. 1 ▶ *c*), or *D*_e_ in transmission mode (Fig. 1 ▶ *e*). The resolution in Fig. 1 ▶(*c*) now depends on the size of the slit at *S*_o_, whereas that in Fig. 1 ▶(*e*) relies on the exactness of the curvature of the collimating crystal. High resolution is relatively straightforward to achieve in reflection mode; however, in transmission mode this is more problematic, because of the difficulty in bending a single crystal to such precision. Other options in high resolution also include monochromators in the diffracted beam (Lang, 1956 ▶).

In all cases of improving the resolution, the instrument becomes significantly larger. Ideally, we would like to achieve high resolution with good intensity, use a reasonable-sized sample and keep the instrument small. The concept described in this article has these features and overcomes many of the issues raised above. The size of the sample does not define the resolution, the Cu *K*α_1_ radiation is isolated and the incident beam on the sample is sufficiently divergent to give good scattering from a random distribution of crystallites. The resolution can match that of the best laboratory instrumentation available, and the instrument can be made very compact without the need for scanning.

### 1.3. Summary of factors that limit the resolution and speed of data collection

The criteria that define the resolution and are associated with the incoming and outgoing beams from a sample are listed below:

(*a*) Wavelength dispersion, *i.e.* the broadening of the profiles because of the distribution of wavelengths incident on the sample that are scattered by the sample. This gives an effective elongation of the scattering vector magnitude
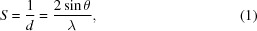
where λ (= 1/|**k**|) is the wavelength and θ is half the scattering angle; then
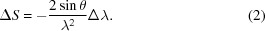



(*b*) Beam divergence in the scattering plane, *i.e.* the divergent rays within the incident beam that impinge on crystallites will scatter these rays, **k**_H_, in a fixed relationship to the incident ray direction, **k**_0_, given by the angle 2θ for a given wavelength, equation (1), thus giving a spread in the scattered rays leaving the sample and consequently broadening the profile.

(*c*) Axial divergence, *i.e.* the component of the divergence normal to the scattering plane. Axial divergence gives the opportunity for the rays to be scattered out of the scattering plane, and because of the fixed relationship of the scattered rays to the incident rays, this adds a projection effect. The broadening is most obvious at the low-angle side of the recorded peaks and for small scattering angles. This broadening also occurs on the high-angle side of large scattering angles.

(*d*) The finite size of the beam. This has particular relevance to methods that collect data by position, *e.g.* position-sensitive detectors, where the volume from which the rays are scattered will add to the beam width. If the configuration relies on focusing geometry then the finite size contribution to the resolution will be determined by the size of the X-ray source, the incident beam slit and the slit in front of the detector, or the quality of the focus in the plane of a position-sensitive detector.

All these aspects contribute to varying degrees and most approaches have been exploited, *e.g.* monochromation to reduce the wavelength dispersion, focusing methods to reduce the influence of beam divergence, Soller slits to reduce the axial divergence (Soller, 1924 ▶) and reducing the sample size in transmission geometry. It is also clear from the discussion above that the highest resolution is most easily obtained with focusing geometry in scanning mode; however, this requires the data to be collected in series, step by step. To improve the speed of the scanning, a position-sensitive multiple-strip detector can be used, such that the intensity scattered at a specific angle is measured as many times as there are strips, with an increase in peak spreading due to the flat detector effect, which is discussed in a later section.

## 2. Compact geometry: theory, concept and configuration

Ideally, we would like to collect as much data as possible in parallel and move away from the complications of focusing geometries, as these require higher tolerances for smaller samples and detector radii. An ideal solution is to create a beam that is monochromatic, small and intense, with sufficient beam divergence to bring enough crystallites into a position where they can scatter; the data can then be collected in parallel with a position-sensitive detector. The incident beam will, therefore, define the scattering area rather than the sample size. In this geometry, the full sample volume is also defined by the sample thickness. If the beam is sufficiently small then focusing geometry is unnecessary to achieve high resolution in a very compact geometry, provided that the wavelength dispersion is minimized. Since this does not rely on focusing geometry, planar position-sensitive detectors can be used rather than elaborately curved configurations. To achieve these requirements we have exploited the concept given by Fewster (2005 ▶), so that a small monochromatic beam could be created.

**Figure 2:**
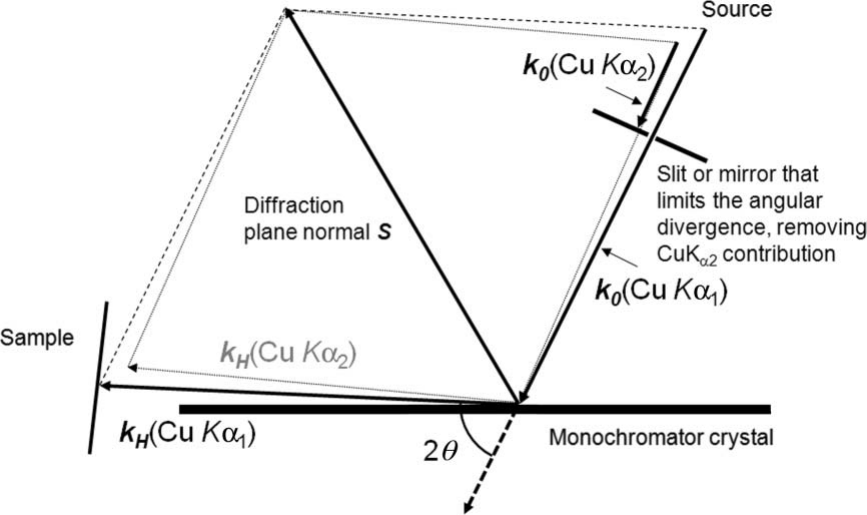
The geometry of grazing exit diffraction, indicating how a combination of the monochromator crystal and a restriction of the incident divergence can isolate the Cu *K*α_1_ line. The Cu *K*α_1_ and Cu *K*α_2_ beam paths in the figure represent those required to satisfy the scattering condition from the monochromator.

In the setup described by Fewster (2005 ▶), a divergent incident beam scattered from crystal planes of a nearly perfect single-crystal sample, such that it emerges at grazing exit, will create a very small but more divergent exit beam. The approach of using low angle of exit has been exploited by Frankuchen (1937 ▶) and Evans *et al.* (1948 ▶) to obtain intensity enhancement through beam compression and surface roughening. However, it is the modification to the divergence that comes from the conservation of energy, as described by dynamical theory, that is of most interest in the present application. If the divergence of the incident beam is restricted by a slit or an X-ray mirror and the monochromator crystal is rotated, then the wavelength range can be restricted and controlled, such that the *K*α_1_ spectral line can be isolated from the *K*α_1_–*K*α_2_ doublet (Fig. 2 ▶). Any incident ray can be associated with a scattered ray and, therefore, a small change in the incident angle, for example observing subtle thickness fringes in a perfect epitaxically grown multilayer sample, will produce well separated rays in the scattered beam. This is a very simple way of creating a high-resolution diffractometer for epitaxial layer samples, since all the rays appear to emanate from a very small region of the sample when viewed at grazing exit. This small, but divergent, Cu *K*α_1_ radiation beam is ideal for powder diffraction. The exact nature of the beam, *i.e.* wavelength distribution, size and divergence, can be estimated through simulation of the whole system.

### 2.1. Details of the geometry

**Figure 3:**
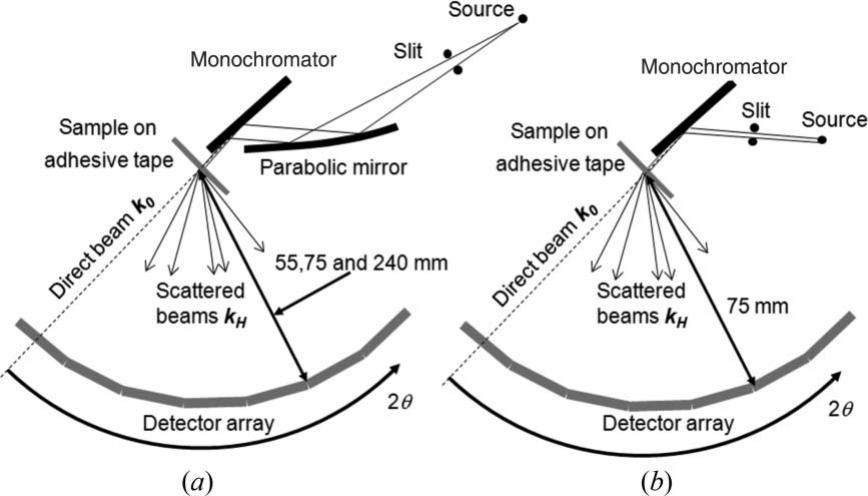
(*a*) The geometry of the compact diffractometer and the dimensions used in this study, shown with a full array of detectors that is equivalent to a single detector at several locations. This tiling configuration would then compare with other curved position-sensitive detectors. (*b*) The geometry of the diffractometer in its most compact form with no mirror.

**Figure 4:**
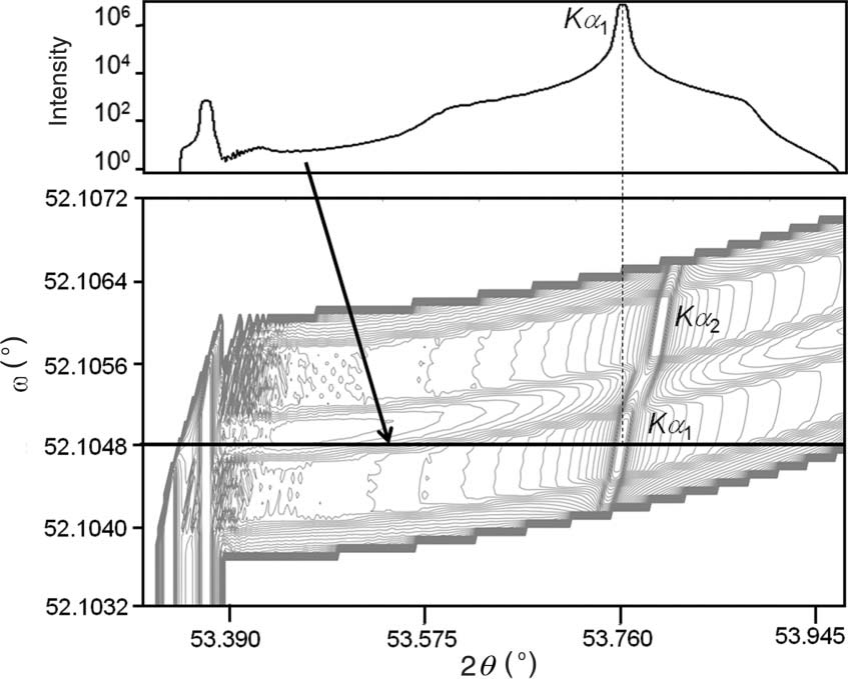
The calculated intensity distribution in diffraction space for the monochromator as it is rocked in ω and 2θ (lower view). The peaks for the Cu *K*α_1_ and Cu *K*α_2_ lines are clear; the abrupt drop in intensity, peaks and intensity fluctuations at the low 2θ values are associated with scattering close to the grazing exit critical angle. For the experiments the monochromator is set in ω to the *K*α_1_ peak, and this gives the divergence profile as in the upper view. The calculation is performed in reciprocal space coordinates and then converted to diffraction space for the angular coordinates.

For the purpose of creating a small beam of a suitable divergence to study powder samples, the 113 reflection from a single crystal of GaAs with a (001) surface orientation was used, in combination with an X-ray mirror and a line focus source (Fig. 3 ▶ *a*). This setup has also been operated in a simpler form without a mirror, with a consequential reduction in intensity (∼×10) and higher resolution, but making it more compact (Fig. 3 ▶ *b*). Fig. 4 ▶ illustrates a simulation of the diffraction space map based on the configuration with the mirror, which defines the divergence, whereas the configuration shown in Fig. 3 ▶(*b*) has a divergence defined by the slit as in Fig. 2 ▶. The coordinates in Fig. 4 ▶ represent the variation of the incident angle on the GaAs crystal (ω) *versus* the scattered angle from the GaAs crystal (2θ). The simulation is based on ray tracing, described by Fewster (2005 ▶), and dynamical theory, including all four beams generated from tie points on the dispersion surface (Holý & Fewster, 2003 ▶). This is necessary since the conventional two-beam dynamical theory does not account for the strong influence of the internal specular beam in suppressing the intensity below the critical exit angle. It is clear that a small angular spread in ω will create a large angular spread in 2θ; from this we can determine the angular spread of the exit beam from the GaAs crystal to be 0.011°. This is the divergence of the beam from this monochromator. The beam leaving the mirror is 1.2 mm wide, has a divergence of ∼0.04°, and includes a spectral distribution that covers both Cu *K*α_1_ and Cu *K*α_2_; the exact magnitude of this divergence is not relevant since the subsequent divergence acceptance of the GaAs collimating crystal is much less than this. The axial divergence is calculated from the source, through the mirror and onto the sample.

**Figure 5:**
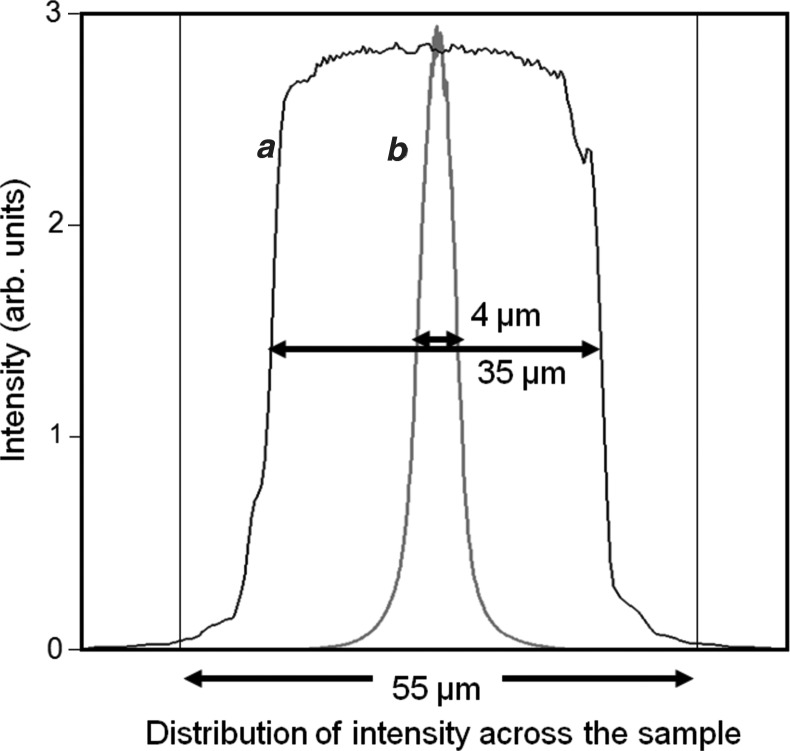
The calculated distributions of intensity arriving at the sample in the scattering plane; curve *a* with the mirror, and with the sample 20 mm from the monochromator crystal, and curve *b* without a mirror but with an 80 µm slit and with the sample 20 mm from the monochromator crystal. The noisiness in the simulation comes from the digital nature of the calculation.

The powder sample was placed so that its distance from the point at which the beam exits the GaAs crystal was ∼20 mm. Calculation gives the distribution of the intensity at the powder sample position, as shown in Fig. 5 ▶, as well as that for the configuration without the mirror. The powder sample is captured on some adhesive tape and placed normal to the beam. The data sets presented in this article were collected with an area detector, for sample-to-detector radii of 55, 75 and 240 mm, depending on the application. 55 mm represents the minimum radius possible without collisions in this setup, 75 mm is a suitable compromise for intensity and resolution, and 240 mm is used for the highest resolution experiments. Immediately in front of the detector, a 0.02 rad Soller slit has been used to remove the cross-fire from an otherwise unrestricted axial divergence. Various Soller slit sizes (0.08, 0.04 and 0.02 rad) have been used, and although the latter results in a greater loss of intensity, the signal/noise ratio is superior. For the very highest resolution at low scattering angles, the smaller Soller slits are necessary; however, for rapid measurements 0.04 or 0.08 rad Soller slits boost the peak intensity in the examples given, by ∼×2 and ×3 and with a 10–20% increase in peak width at 25° (2θ), with respect to measurements using 0.02 rad Soller slits.

### 2.2. The sample and sample mounting

**Figure 6:**
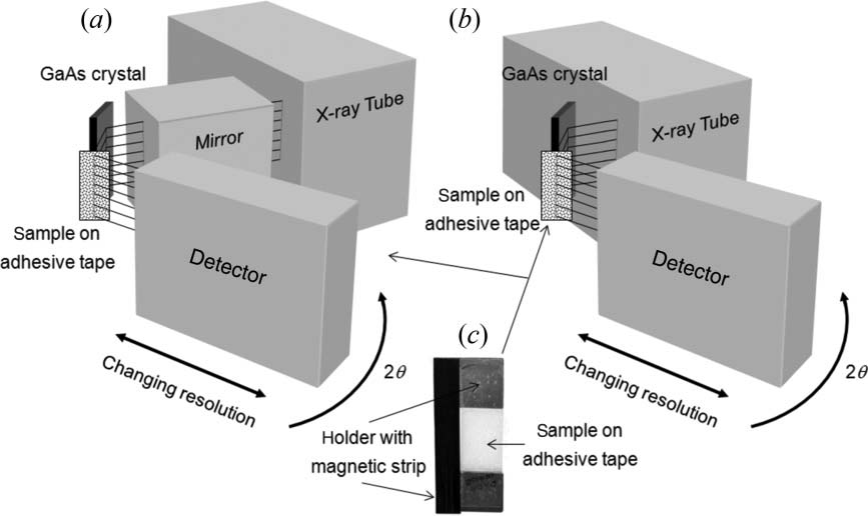
A schematic representation of the arrangement (*a*) as used in this study and the configuration (*b*) in the most compact form (see Fig. 3 ▶ *b*). (*c*) The U-shaped mount comprising the sample captured on adhesive tape and the magnetic strip for rapid mounting. No sample alignment is required since the sampled volume is defined by the incident beam.

To maintain a small volume of sample for achieving high resolution, the powder under study was collected on adhesive tape, producing a layer of sample that was approximately one crystallite (3.5 µm) thick when using LaB_6_ (NIST 660a standard, with a crystallite size distribution from 2 to 5 µm). This gave a potential scattering area of ∼40 × 3.5 µm (∼3 × 3.5 µm without the mirror) in the scattering plane and a beam 15 mm high. The intensity was measured in these experiments with a photon counting solid-state pixel detector (PIXcel from PANalytical), with pixel dimensions of 55 × 55 µm positioned at a radius of 55 mm up to 240 mm. There are 256 × 256 pixels and this equates to an angular range of 10.75° in 2θ at 75 mm radius. The signal from the pixels normal to the scattering plane is integrated into strips. The data have been collected using a stationary detector in this mode, whilst the sample is rocked or rotated. A schematic of the configuration used in this study is given in Fig. 6 ▶(*a*) and the sample mount in Fig. 6 ▶(*c*). A schematic of the configuration with no mirror is given in Fig. 6 ▶(*b*).

With this configuration, we can observe the incident beam at the 2θ position directly; the intensity is ∼93 Mcounts s^−1^, the wavelength is pure Cu *K*α_1_ and the beam is contained within one column of pixels (<0.042°). This width is composed of a beam of 35 µm with an angular divergence of 0.011°, as mentioned above. Because the pixel size defines our angular resolution, and the scattered beam can be narrower than this width, the detector response can differ for various scenarios; for example, when a photon arrives close to the edge of a pixel, the peak height, shape and width will be modified. However, for the scope of this paper, we shall consider at this stage only the influence of the pixel size and the apparent resolution, and concentrate on the practical aspects of data collection and performance. It is important to also understand at this stage that the scattering in a powder diffraction pattern is almost entirely composed of intersections of the beam with the tails of the scattering from crystallites, rather than within the width of the Bragg peaks (P. Fewster, in preparation; Fewster & Andrew, 1999 ▶; Fewster, 2000 ▶): if this were not the case then we would not expect any observable scattering from so few crystallites. The scattering is more to do with the divergence of the beam that each crystallite experiences and not the spread in divergence across the whole sample. Thus each crystallite of say a few micrometres in combination with a distant X-ray source of say 40 µm will, effectively, create a high-resolution scattering profile. Hence, whether the beam has a spread of divergences is not important, except that it may illuminate a bigger area and more crystallites. This latter point gives a method for estimating the scaling factor for the pattern, compared with the conventional Bragg–Brentano geometry.

## 3. Intensity comparison with current methods

In this section, aspects of the new configuration (Fig. 3 ▶ *a*) are compared with the Bragg–Brentano configuration (Fig. 1 ▶ *c*), in typical arrangements using slits, graphite and Ge crystal monochromators. As an order of magnitude calculation, the following assumptions are made: the same line focus size is used for the two geometries, the distribution of orientations of the crystallites is similar and the absorption depth in reflection geometry is comparable to the layer of powder on the tape. The linear absorption coefficient of LaB_6_ is 1098 cm^−1^ for Cu *K*α radiation, so as a solid mass the penetration is ∼1 µm, defined as the 1/*e* point.

Initially, an estimation of the number of crystallites involved in scattering is derived for the two geometries. This number of crystallites for the two geometries gives an estimate of the scaling parameter, since the probability of scattering for each crystallite is similar, as briefly outlined above.

### 3.1. The number of crystallites illuminated in the compact geometry

In the compact geometry, the direct beam through the sample can be measured by comparing the intensity without the sample (93 Mcounts s^−1^), with the sample mounting tape (89 Mcounts s^−1^) and with the sample and sample mounting tape (61 Mcounts s^−1^). Thus, the tape absorbs 5.76% of the beam and much of the beam passes through the sample. Suppose we assume that there is a single layer of LaB_6_, spherical crystallites of 3.5 µm diameter and full coverage in a hexagonal close-packed arrangement; then there will be gaps representing 0.09 of the area. The absorption is then calculated from the path lengths through different parts of the spheres and this will achieve an intensity of 0.14 of the original beam. From the results, it can be seen that the sample does not fully cover the tape. The calculation was then modified by changing the coverage until the ratio of the remaining intensity compared with that from the direct beam (0.66) was matched. This ratio related to a coverage of 38%. Where there is compete coverage of an area of 15 mm × 40 µm (representing the imprint of the direct beam on the sample) it will contain a maximum of ∼62 300 crystallites {this is based on 15 000 × 40/[π(3.5/2)^2^]}. Hence for 38% coverage this reduces to ∼24 000 crystallites. This estimation does not take account of the loss in intensity from scattering (to do this the total intensity scattered, integrated over 4π steradians, should be subtracted) so the actual coverage will be slightly larger than 38%, thus increasing the actual number of crystallites in the beam above 24 000. When the mirror is removed, the number of crystallites drops to 2000 and the incident intensity by an order of magnitude; however, the peaks are still all observable.

### 3.2. The number of crystallites illuminated in the Bragg–Brentano geometry

In the Bragg–Brentano geometry, because of the high absorption in the LaB_6_ crystallites, the intensity cannot easily be transmitted through a crystallite and then scattered. For a perfectly prepared hexagonal close-packed sample, an area of 15 × 10 mm would contain ∼15 600 000 crystallites. This value is probably an upper limit and does not take into account the strong angular dependence if the slits are fixed at specific values. If 15 × 10 mm is the dimension at 20° in 2θ, this reduces to 15 × 4.2 mm at 50° and 15 × 2.3 mm at 100°, giving 6 540 000 and 3 590 000 crystallites illuminated, respectively. For transparent samples, these figures are not precise since the penetration and therefore the number crystallites accessible for scattering will be greater.

### 3.3. Intensity calculations

The next important parameter is the intensity arriving at the sample. This can only be estimated properly by ray tracing, but for an order of magnitude calculation the following aspects are considered. Losses due to axial divergence and air scatter increase with beam path length. The X-ray mirror in the compact geometry boosts the intensity, although the GaAs crystal decreases the intensity. Unfortunately there is no easy way of measuring the incident intensity with the Bragg–Brentano geometry, because the beam from the source is continuously diverging. For comparison we shall consider the simplest form of this geometry with no primary-beam monochromator in the first instance. A typical radius is 240 mm, although our comparison was conducted on an instrument with a radius of 320 mm. The Bragg–Brentano geometry is considered first.

### 3.4. Intensity available for scattering in the Bragg–Brentano geometry

The instrument function varies rapidly with angle and therefore an estimate of the change in intensity, due to the reduction in divergence, is required. As the beam propagates from the source it spreads through circumferential dispersion, with a loss in intensity proportional to α/π, where α is the divergence angle accepted by the sample from the source when no focusing is involved. If we have a 10 mm-long sample and incident (divergence) and receiving (acceptance) angular slits of 0.25°, then provided the measured peaks are above 16.5° in 2θ, the angular acceptance is unchanged and restricted to 0.25°. If the sample is 8 mm long, then for the sample to make full use of this 0.25° divergence requires an angle of 20° in 2θ or above. For a 4 mm sample this becomes 0.126° at 20°, and becomes independent for angles of 40° and above. These divergence angles define the beam intensity for scattering when they are less than those of the divergence slit, and result from the finite size of the sample viewed from the source. The axial divergence losses are less dramatic when Soller slits are used, *i.e.* we are limiting the axial divergence so that losses through circumferential dispersion with distance are reduced. Soller slits are used in both geometries, so we assume these losses are comparable. We can conclude that the intensity reduction from the source to the sample is (0.25)/180 × 0.66 = 9.167 × 10^−4^ for a radius of 240 mm or 7.97 × 10^−4^ for a radius of 320 mm (the factor 0.66 is the proportion of the X-ray beam not absorbed in air). The remaining losses relate to the probability of scattering at the angle of interest for this so-called symmetric geometry, if we assume that these scattered beams are accepted by the receiving slit and detector. Also, in this fixed divergence geometry, the number of crystallites illuminated reduces with increasing angle 2θ.

### 3.5. Intensity available for scattering in the compact geometry

For the compact geometry, the determining aspects are the angular acceptance of the X-ray mirror (0.8°) and losses on reflection (reflectivity is ∼65%), together giving a reduction factor of (0.8/180) × 0.65 = 2.89 × 10^−3^. This beam is then converted into one with a divergence of 0.04°, so the divergence losses with distance are minimal and the intensity increase within this angular range is ×13. Capturing this divergence spread improves the available intensity factor to 3.76 × 10^−2^. The angular acceptance of the GaAs crystal is small, which has the advantage that the sensitivity to angular rotation is small (it is effectively selecting an angular spread of 0.0004° out of the 0.04° from the mirror); this brings the reduction in the intensity to 3.76 × 10^−4^. This angular acceptance is such that we can easily separate the *K*α_1_ radiation from the spectrum. The reflectivity of the GaAs 113 reflection is ∼71% in the σ polarization and 58% in the π polarization, giving an overall reflectivity of 66%: reducing this value to 2.48 × 10^−4^. The influence of the polarization losses from the mirror is minimal. Because of the small exit angle from the GaAs crystal [(001) surface with the 113 reflection], the scattering appears from a small projected area at the sample. From dynamical theory, the scattered beam from the GaAs crystal is more divergent than the accepted incident beam from the mirror, and hence the beam on the powder sample has an angular spread of 0.0106° coming from an apparent source of 35 µm. All this angular spread is seen by the sample and, therefore, there are no losses apart from air absorption. Considering all these influences and the losses due to air absorption (∼10% cm^−1^), then the reduction in the beam intensity from source to sample is 2.48 × 10^−4^ × 0.89 ≃ 2.2 × 10^−4^, apart from losses due to the reduced wavelength band pass. Again we can assume that the remainder of the intensity is modified by the Soller slits and the detector response, and this is not significantly different for the two geometries, although the air absorption and axial losses are considerably less in the compact geometry. The wavelength band pass is controlled by the divergence of the beam onto the GaAs crystal, whereas the Bragg–Brentano geometry has an unrestricted wavelength contribution close to the *K*α doublet. The wavelength contribution in the compact geometry can be visualized from Fig. 4 ▶. The line of constant wavelength follows the curve of the contours, and the extracted distribution follows the line for ω = 52.1048° with an intensity given in the profile above, when set to the highest peak intensity corresponding to the *K*α_1_ characteristic wavelength. The comparison below will be based on the peak intensity rather than the integrated intensity, so it is the *K*α_1_ peak height that is important and not the full spectral distribution.

### 3.6. Comparison of the intensity and data collection for the two geometries

The intensity ratio of the compact diffractometer (75 mm radius) to the Bragg–Brentano diffractometer (240 mm radius) would be (2.2 × 10^−4^)/(9.167 × 10^−4^) = 0.241, if the whole beam was used in each case. The experimental comparison with this geometry was carried out using a 320 mm radius and with a graphite monochromator to improve the background and suppress the Cu *K*β line, which has a reflectivity of ∼33% and polarization factor of 0.97. Thus the intensity ratio for LaB_6_, where the compact diffractometer has 38% coverage, is 0.324 [= (2.2 × 10^−4^)/(7.97 × 10^−4^) × 3/0.97 × 0.38]. For this particular comparison, another factor in the configurations is that the detector in the Bragg–Brentano diffractometer has a large axial beam acceptance, 27 mm compared with 14 mm for the compact diffractometer. This gives an intensity ratio of the compact diffractometer (75 mm radius) to the Bragg–Brentano diffractometer (320 mm radius) of 0.162.

**Figure 7:**
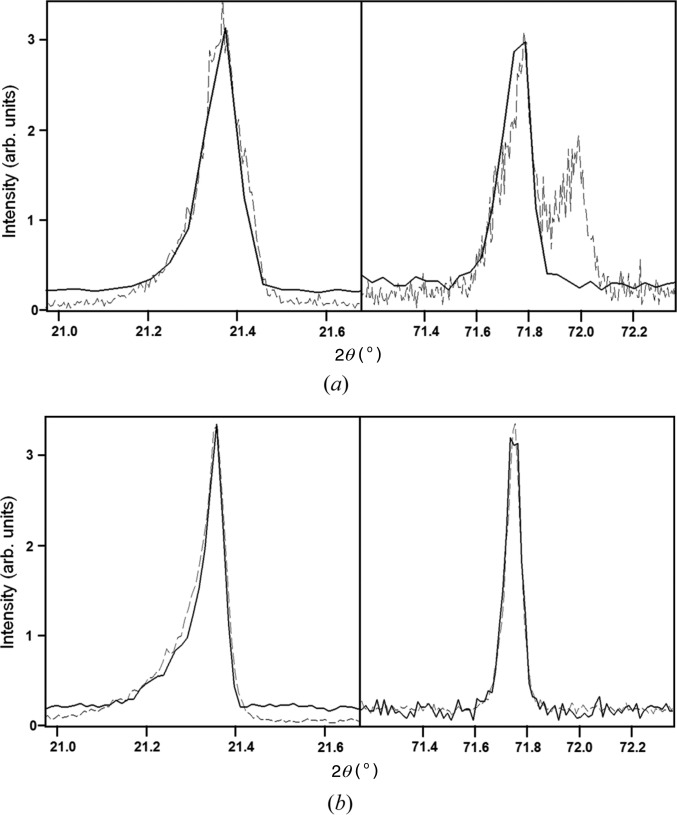
(*a*) A comparison of the profile from the compact diffractometer (radius 75 mm) (continuous line) and the Bragg–Brentano diffractometer (radius 240 mm) (dotted line) at two different 2θ angles. (*b*) A comparison of the profile from the compact diffractometer (radius 240 mm) (continuous line) and the Bragg–Brentano diffractometer, with a focusing monochromator (radius 240 mm) (dotted line), at two different 2θ angles.

A comparison of the profiles for the compact geometry and the Bragg–Brentano geometry, the latter with a graphite monochromator, is given in Fig. 7 ▶(*a*). The experimental ratio 0.145 (2) compares well with the calculated intensity ratios (Table 1 ▶). The spread represents a few repetitions of the measurements. For the compact diffractometer, through simulation of the ω alignment scan, *i.e.* integration of the intensity in 2θ, it can be shown that the intensity is not significantly modified by having a bent GaAs crystal; for example a 10 m radius of curvature reduces the intensity by ∼10%. Defects within the GaAs crystal, though, will reduce the reflectivity. All these effects can influence the performance.

### 3.7. Comparison with diffractometers using Cu *K*α_1_ monochromators

To make a more direct comparison with a diffractometer that isolates the *K*α_1_ component, the Bragg–Brentano geometry incorporating a bent Ge crystal as a primary-beam monochromator is considered. The reflection losses arise from 93% reflectivity for a perfect bent crystal, although in reality because of the difficulties in bending with precision within the intrinsic scattering angle, the reflectivity could be considerably less, and addition of path length losses will result in only 66% of the intensity reaching the incident point on the focusing circle, *S*_o_ in Fig. 1 ▶(*c*). A typical intensity remaining at *S*_o_ could be 61% at best. The polarization losses reduce the intensity further by a factor of 0.94, thus making the ratio of the intensities of the compact geometry to the high-resolution monochromated incident beam diffractometer 0.158 [ (2.2 × 10^−4^)/(9.167 × 10^−4^ × 0.61 × 0.94) × 0.38], assuming that the primary-beam monochromator is perfect. The experiment was carried out on an instrument with a large axial detector aperture, making a calculated ratio of 0.070. Experimentally the ratio was determined to be 0.042 (5). Within the nature of the assumptions made, tube flux, alignment, *etc.*, this agreement is quite reasonable. The peak shape comparison between these two geometries is given in Fig. 7 ▶(*b*).

The comparison with transmission geometry is calculated and included in Table 1 ▶, for two standard capillaries of 350 and 700 µm. These are only approximate and based on similar incident and reflected beam path lengths of 240 mm, which will vary with the degree of asymmetry used for the bent Ge 111 monochromator crystal. It is also assumed that the accepted angular spread at the detector from the capillary sample gave an upper value of the circumferential divergence from the source to the detector. More precise calculations would have to be specific to the exact geometry and quality of the curvature of the Ge crystal, sample absorption, compaction *etc*. So these figures should not be taken too literally.

### 3.8. Speed improvement with parallel data collection

As described in the *Introduction* [Sec sec1], the data collection for maintaining a constant scattered beam path length in focusing geometry is sequential. Suppose the data are collected to achieve the same counting statistics (equivalent total numbers of counts) with a single position-sensitive detector, used in scanning mode for the Bragg–Brentano geometry and the static mode for the compact geometry. The data collection in the former is usually performed on a 240 mm radius and with a 55 µm detector strip; the subtended step angle is 0.0131° and this, therefore, is the optimum step size. For the compact diffractometer with a radius of 75 mm, the step size is 0.042°. For both, it is assumed that there are 256 strips, giving angular captures of 3.36 and 10.75°, respectively. Comparing with the Bragg–Brentano geometry with a receiving slit and steps of 0.042° and a 10.75° range, the compact diffractometer is ×61 faster (256 × 0.241) in the worst case (no filtering or monochromation in the Bragg–Brentano geometry). With a diffracted beam graphite monochromator this jumps to ×189 faster. For the comparison with a Bragg–Brentano diffractometer with a diffracted beam monochromator and radius of 320 mm and larger aperture, the improvement is ×42.

If multiple counting during scanning is used for the Bragg–Brentano geometry with a 240 mm radius then these ratios become ×1.25 faster (17.47/3.36 × 0.241, 17.47 represents the actual angle needed to be scanned for a 10.75° range^1^), ×3.75 when a graphite monochromator is used, and ×0.44 if a perfect primary monochromator is available and a large axial detector aperture used. So despite the very small sample volumes of the compact geometry, the performance is comparable, in terms of accumulated counts, to that of the Bragg–Brentano configuration used in multistrip mode when collecting data over an arc of ∼10° in 2θ. However the scattering angle range of the compact geometry can be scaled to 10 × 10.75° (107.5°) or more with no increase in data collection time by tiling the detectors (Fig. 3 ▶). The other geometries scale linearly and therefore over typical data collection ranges the compact geometry can be made considerably faster (see Table 1 ▶). Another advantage of the compact geometry is that the data can be observed during collection, to inspect how the whole profile is emerging; this has the clear advantage of being able to stop data collection when the peaks are resolved and have statistical significance.

## 4. Data collection and analysis

This section concentrates on the specific aspects associated with collecting data with the compact diffractometer and the corrections that need to be considered.

### 4.1. Background intensity

An important aspect of powder diffractometry is the minimization of artefacts associated with residual scatter from slits, sample mounting *etc*. In fact, any contribution to the profile that can lead to wrong interpretation has to be removed. With conventional geometries these have been covered in various textbooks, *e.g.* Klug & Alexander (1974 ▶) and Jenkins & Snyder (1996 ▶). There are other contributions to the background from the wavelength distribution scattering from the sample, fluorescence from the sample and non-crystalline contributions. These effects are not considered here as background, because they contain sample information; our main concern is those contributions that are associated with the method. Scattering from the sample mount can obscure information and this requires considerable care in the Bragg–Brentano geometry because of the very large beam used. In the compact geometry there will be a very weak amorphous contribution from the tape and this can be subtracted from the composite profile if necessary. The contribution from imperfect slits is not easily quantified in the Bragg–Brentano geometry, but hopefully should be consistent and removed during background stripping. This is a significant advantage of this compact geometry in that the background can be measured without the sample, *e.g.* contributions from fluorescence of GaAs and the sample mounting tape. Also the whole shape of the direct beam can be measured, so that the contribution from the sample – the amorphous phases, the crystalline phases and the microstructure – can be isolated rather easily.

### 4.2. The compact geometry: data interpretation considerations

By the very nature of compact geometry, small size and high resolution, it is important to know the sensitive parameters that can lead to misinterpretation. The general alignment of the instrument requires that the line-shaped probing beam on the sample is aligned parallel to the pixel strips in the detector, otherwise there is a spreading of the peak profiles. This is a fairly straightforward process, so will not be covered in this article. Some effects though are systematic, for example, the flat detector correction, and some are more method based, for example, accurate setting of the sample in the centre of the detector circle.

#### 4.2.1. The flat detector correction

It should be noted that, since the detector is flat and does not follow the circumference precisely, the captured profile of the 2θ arc does not relate directly to a linear scale. If we assume the detector centre is accurately perpendicular to the centre of the sample scattering position, then any beam scattered at an angle of Δ2θ from this centre position will be detected at a position further from centre by
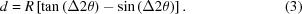
For the smallest radius, *R*, used of 55 mm, this gives a displacement at the outer edge of the detector of 0.044 mm, which is nearly one pixel width, so this is used to correct the data angles. This is a very small deviation from linearity, *i.e.* a maximum of 0.046°. Because the scattered beam is not impinging perpendicular to the detector surface, the beam projection will be expanded from *p* to 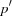, given by
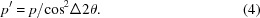
Hence for the dimension used, the edges of the detector will expand a beam that occupies exactly one pixel (55 µm) at the centre, to 55.83 µm at the edge. Clearly this beam expansion is not a serious concern.

#### 4.2.2. Sample displacement errors

**Figure 8:**
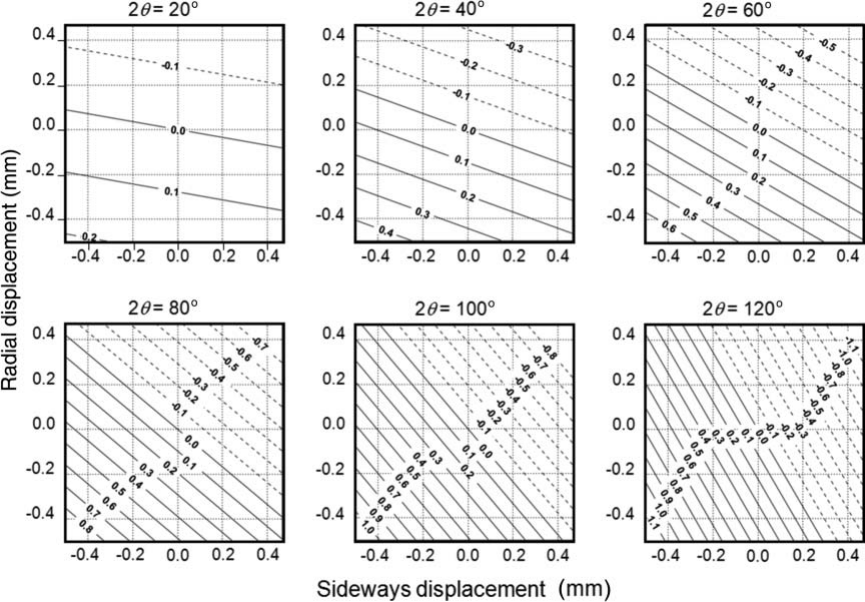
The influence of sample position errors with respect to the 2θ axis. The *x* and *y* displacements of the sample with respect to the 2θ axis are normal and parallel to the incident beam, and are a function of the scattering angle.

The position of the incident beam on the sample and its relationship to the rotation axis of the detector is perhaps the least well defined parameter in this configuration. Any error in the powder sampling position and the axis of the detector arm will result in a difference between the actual and measured 2θ values. If the sample is displaced with coordinates *x*, *y*, where *x* is the distance perpendicular to the incident beam (sideways displacement) and *y* that along the incident beam (radial displacement), then for an instrument with a radius *R*, the correct angle, 2θ_a_, is related to the measured angle, 2θ_m_, by 
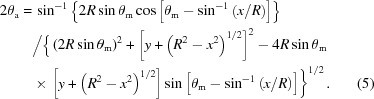
The differences in measured and correct angle can be determined, and are clearly a function of the position on the profile. The sensitivity of the error to displacements at small angles is low; however, as the angle increases towards 90°, the sensitivity increases (Fig. 8 ▶). It is clear that the greatest sensitivity is with *y*, until 2θ_m_ = 90°, when it becomes equivalent and then the greatest sensitivity is in *x*. For a radius of 55 mm, and for the measured scattered beam not to be displaced outside its own half-width of 0.05°, the maximum error in *x* and *y* is 50 µm, for scattering angles up to 90°. For data collection at lower angles this tolerance is less, *e.g.* ∼70 µm in *y* and 200 µm in *x* at 45°, and at 20° the tolerance is up to 120 µm in *y* and 600 µm in *x*.

Clearly, the mechanical tolerances need to be very high to achieve exact peak positions. However, because of the non­linear influence of the displacement errors it is possible to include a systematic correction parameter that can be introduced, if necessary, into the analysis, *e.g.* for phase identification or lattice parameter determination.

### 4.3. The influence of the detector resolution on the peak shape

**Figure 9:**
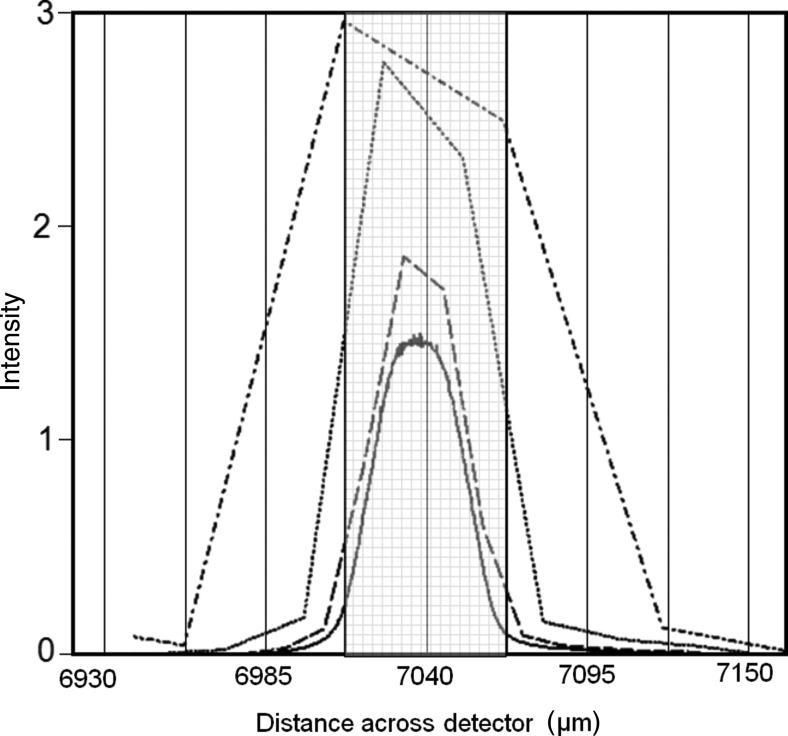
The calculated distribution of intensity impinging on the detector at the direct beam position (2θ = 0°) for the configuration of Fig. 3 ▶(*a*); this can be compared with Fig. 5 ▶ to illustrate the very small divergence in the beam (continuous line). These calculations are for different pixel strip dimensions: dash–dotted line represents the data from a 55 µm pixel (FWHM = 0.101° 2θ) [this pixel dimension (grey region) represents a width of 0.0547° 2θ for the minimum radius used of 55 mm, *i.e.* the most extreme case], dotted line 27.5 µm pixel (FWHM = 0.0535° 2θ), dashed line 13.75 µm pixel (FWHM = 0.0371° 2θ), solid line 6.875 µm pixel (FWHM = 0.0334° 2θ).

As described earlier, the beam dimension on the sample is 35 µm at 2θ = 0°, which diverges to 36 µm at the detector for a 55 mm radius and a few micrometres greater at 75 mm. The scattered beam therefore can be significantly less than the dimension of the pixel strips, and the pixel size limits the resolution. The ray-tracing calculation yields the distribution of intensity at the sample (Fig. 5 ▶) and will produce a distribution of intensity at the detector at 2θ = 0° given in Fig. 9 ▶. The narrow central curve in Fig. 9 ▶ is effectively a measure of the intensity with very narrow pixel strips; however, in practice these are finite, and for the detector used in this experiment, the strips are 55 µm wide and the peak is effectively broadened. This is illustrated in Fig. 9 ▶ for several pixel strip sizes, which indicates how the real resolution of the instrument appears. The difference in intensity of the various curves represents the true intensity, since the larger pixel sizes will improve the counts recorded per pixel. From this it is clear to see that, despite the resolution of the detector, the FWHM appears as ∼0.11°. However, almost all the intensity can be contained within one pixel strip, depending on the position of the detector with respect to the beam, so the width of 0.11° is purely a consequence of the linear interpolation associated with the drawing of the profile. A histogram would perhaps be more representative and produce a width closer to 0.05°.

Another aspect that influences the resolution is that the diffracted beam width, *w*_2θ_, is reduced with scattering angle. This is a pure projection effect because of the nature of the sample cross section (35 × 3.5 µm for 3.5 µm-diameter crystallites of the LaB_6_ standard) in the scattering plane; hence an even distribution of scattering across the sample, *w*_0_, will reduce the parallel contribution to the beam width by
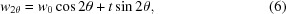
where *t* is the thickness of the sample on the tape. Moving the detector further from the sample makes this contribution less significant and hence the resolution improves.

## 5. Data collection examples and discussion

The aims of this section are as follows: to illustrate how reliable intensity data can be collected very rapidly in terms of sample preparation, mounting and count time; to describe how this configuration can cope with weak scattering, to give an indication of the achievable resolution; and to indicate how dynamic experiments can benefit from this geometry. The emphasis on relatively simple but standard materials enabled direct comparisons to be made with results from conventional geometries.

The compact diffractometer uses a Cu long fine-focus X-ray tube set to 45 kV and 40 mA, a W–Si multilayer mirror, a (001) surface-orientated GaAs single crystal mounted on a goniometer head (for ease of initial alignment) and a PIXcel detector from PANalytical. Because of the low noise of the detector, the results scale with X-ray tube power and the profiles collected at 30 kV and 10 mA have the same dynamic range as those collected at 45 kV and 40 mA, if the data collection time is increased accordingly. The instrument, at a prototype level of development, also shows that even with limited mechanical precision very good results can be obtained. For estimating the data quality, some of the results are compared with those from a PANalytical MRD with fixed slits and a graphite diffracted-beam monochromator.

### 5.1. The compact diffractometer for phase identification

**Figure 10:**
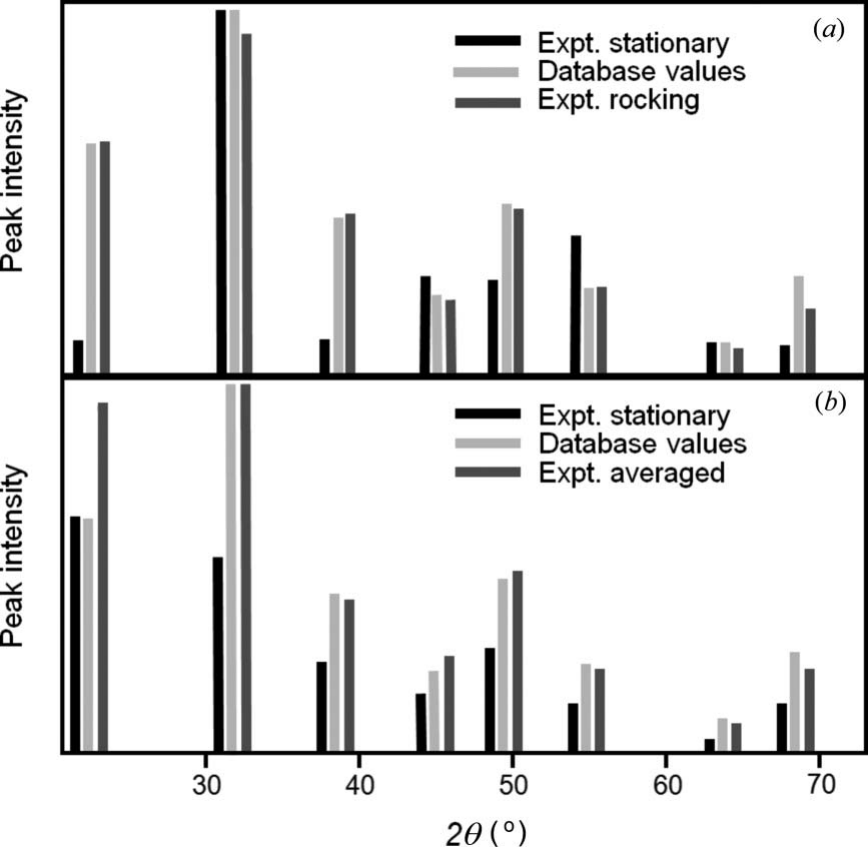
(*a*) A histogram of the scattered intensities collected in five 14.08° segments for LaB_6_ with no sample movement, showing that all the expected peaks appear. When the sample was rocked in the beam the intensities matched those from the database. This is the case for a detector radius of 55 mm. (*b*) A histogram of the scattered peak intensities collected with the Bragg–Brentano geometry, illustrating the same behaviour; all the peaks are shown, but not until the sample is rotated (in this case measured at several azimuths and summed) do they match those in the database. The intensity of the first peak includes a contribution from the Cu *K*α_2_ characteristic line that has increased the peak height. The database used is based on a monochromatic source.

This section serves to emphasize that all the expected peaks could be observed despite the low number of crystallites with this configuration. It will also illustrate how well the intensities matched those from database values. The sample used here was LaB_6_ (NIST 660) because of the good range of peaks and reliable intensities. The data sets were collected with and without rocking the sample, where the rocking movement was perpendicular to the incident beam (Fig. 10 ▶ *a*). They were collected in four 14° sections (radius of 55 mm), counting for 30 s at each of the four positions. The results are compared with those collected with the Bragg–Brentano geometry (Fig. 10 ▶ *b*), in which a diffracted-beam graphite monochromator was used to improve the background and remove the Cu *K*β radiation component. The radius of the instrument was 320 mm, and because the main axes were vertical, the sample was held in place with adhesive tape. This tape gave rise to a broad peak at 18.5°, which is observed in all the experiments. Initially the sample was not rotated.

In Fig. 10 ▶(*a*) all the expected LaB_6_ peaks are observed, and the intensity at each position may vary from that expected when the sample is kept stationary. When the sample is rocked, most of the peak intensities matched the database values within 10%. The results are very similar for the Bragg–Brentano geometry, where again some averaging is required. Measurements are presented in Fig. 10 ▶(*b*) for the Bragg–Brentano geometry with and without sample rotation. The comparison with the database peak heights is also given. It is clear that both methods require some sample movement to give a reasonable estimate of the intensities, and that all the peaks are visible in all the profiles from both methods. Despite the large disparity in numbers of crystallites (∼6 000 000 in the Bragg–Brentano geometry and ∼24 000 in the compact diffractometer geometry), the quality of the intensity data is not degraded in the latter. The highly reproducible intensities achieved with this instrument naturally lead to very high precision when obtaining quantitative phase proportions. The disparity in the Bragg–Brentano peak intensity values, especially for the lowest 2θ peak is a consequence of comparing peak as opposed to integrated intensities. The closeness of the Cu *K*α_2_ and Cu *K*α_1_ components will result in them both adding to the peak intensity at low angles, whereas at higher angles they are resolved and the Cu *K*α_1_ peak intensity is given. This is not the case with the compact diffractometer since only Cu *K*α_1_ is used.

### 5.2. Measuring samples that scatter weakly

**Figure 11:**
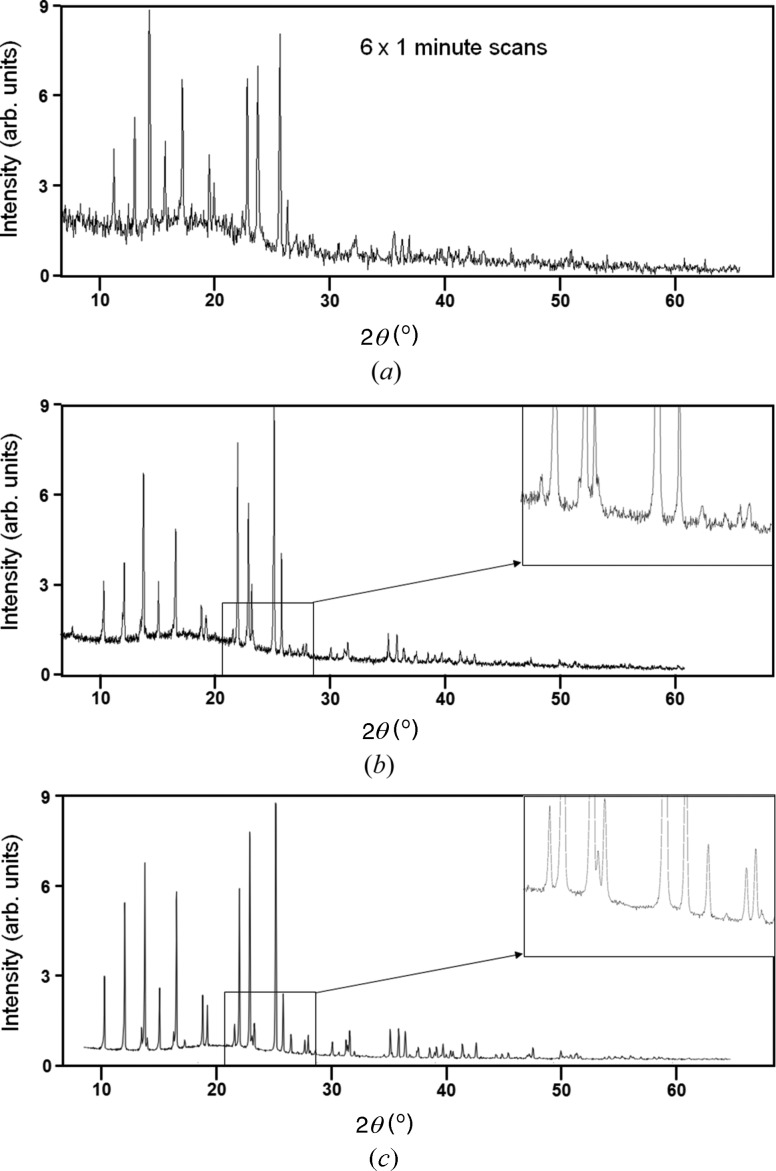
(*a*) The full scattering profile from a crushed paracetamol tablet from 7 to 65° 2θ, with sample rocking, collected in 6 × 10.75° 2θ static data captures of 1 min each. (*b*) The scattering profile collected at a radius of 240 mm, and collected in six 10 min captures using a 256-strip detector, giving a total collection time of 2.7 h. These are raw data, so smoothing would improve this further, as would longer collection times. (*c*) The full scattering profile from paracetamol, collected by Florence *et al.* (2005 ▶), for precision structure determination, 10 s per 0.0145° step in 2θ, equating to 10 h data collection time.

As an example, we used a crushed tablet of paracetamol, obtained from a local store and compared the results with those in the literature. The sample preparation was simply to crush the tablet to create a roughly uniform powder, which was then collected onto some adhesive tape and stuck onto a U-shaped mount that has a magnetic strip to locate it easily onto the rocking mechanism (Fig. 6 ▶ *c*). The sample-to-detector distance was set at 75 mm and the data sets were collected in 10.75° 2θ ranges, counting for 1 min in each position while the sample was rocked perpendicular to the incident beam. The profile is given in Fig. 11 ▶(*a*) and is compared with very carefully collected laboratory data (Fig. 11 ▶ *c*) that required a 10 h scanning time (Florence *et al.*, 2005 ▶). Florence *et al.* used a Ge 111 bent crystal monochromator focused onto the detector; the sample was mounted in a 700 µm capillary on a Bruker AXS D8 Advance goniometer. All the major features are reproduced with the compact diffractometer in a few minutes, although the resolution is slightly poorer partly because of the limited pixel size at this radius and partly because of the strain induced during sample preparation. It must also be recognized that the proportions of packing components such as maize starch, potassium sorbate, talc, stearic acid, povidone and soluble starch are unknown and reduce the quantities of the active components, with a consequential additional noise level.

The profile for paracetamol is highly reproducible and most of the main features can be observed within a few seconds. This could well make this instrument suitable for rapid identification, due to the ease of sample preparation and speed of data collection.

### 5.3. The compact diffractometer for microstructure analysis

The paracetamol data, Fig. 11 ▶(*b*), were collected in 2.7 h, made up of 10 min scans at 16 positions of the detector and at a radius of 240 mm. This compares with the sequential data collection in Fig. 11 ▶(*c*) which took ∼10 h. Clearly, an array of detectors at this radius would allow high-resolution data to be collected very rapidly. The peak widths for this sample were 0.08 (2) over the range captured and are identical to those quoted by Florence *et al.* (2005 ▶).

**Figure 12:**
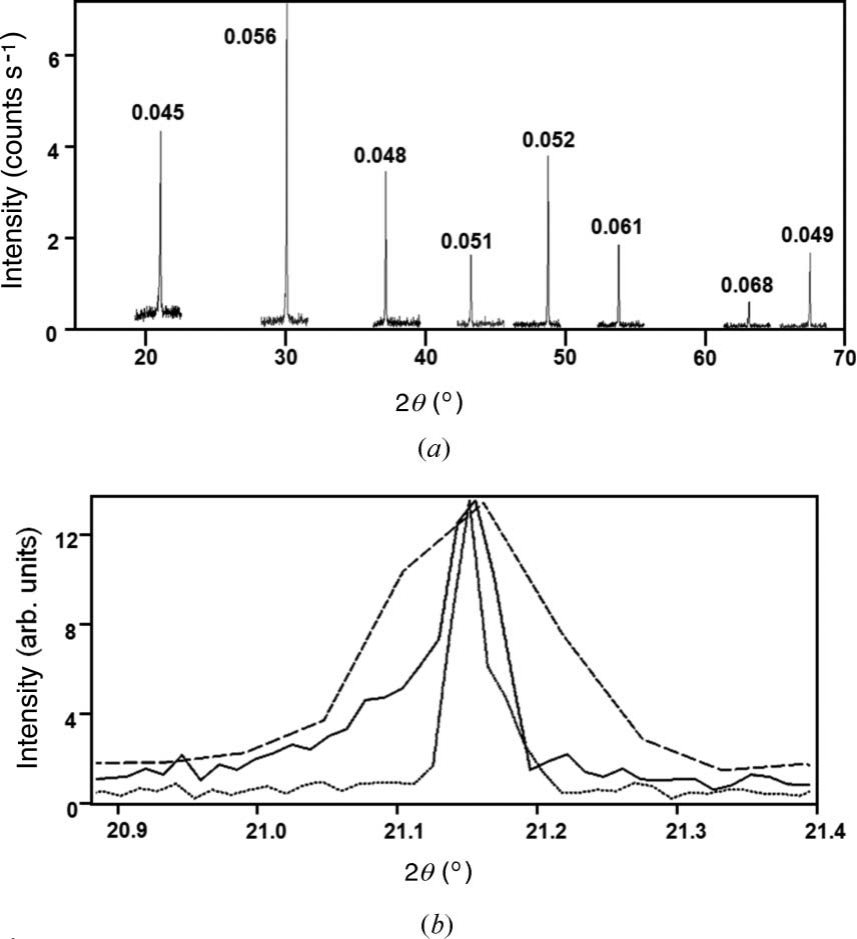
(*a*) The peaks of LaB_6_ in high-resolution mode (radius of 240 mm) with the data collected using a stationary detector at each peak position while rocking the sample. The intensities match those of the database (*cf.* Fig. 10 ▶) and the peaks widths match those expected from theory (see text). (*b*) The profile of 001 LaB_6_ at 55 mm (dashed line) and 240 mm (continuous line), indicating how the resolution is improved, and how without rocking sometimes the peak profile can be very narrow, suggesting that the intrinsic instrumental broadening is very small (dotted line).

As mentioned in the description of the geometry and the modelling of the monochromator performance, the ultimate resolution is ∼0.01°. However, this is limited by the finite size of the beam on the sample (∼35 µm) and the detector pixel size. Moving the detector further away from the sample increases the resolution, as the subtended angle of the detector pixel strip to the sample–detector radius reduces and the sampled size becomes less significant. At a sample-to-detector distance of 55 mm, the 001 profile of LaB_6_ is ∼0.13°, at 110 mm this is reduced to ∼0.079°, and at 240 and 300 mm the width stabilizes at ∼0.05° (Fig. 12 ▶ *a*). These represent the conditions when the sample is rocked, whereas if the sample is stationary these peak widths can vary significantly for the reasons given and discussed by Fewster & Andrew (1999 ▶). An example for LaB_6_ at a radius of 240 mm with a stationary sample is given in Fig. 12 ▶(*b*) showing a peak width of ∼0.026°, although the narrowest profile that has been observed was 0.023°. The maximum instrumental broadening at this radius is ∼0.019°, taking into account the divergence and finite dimensions of the beam when using the mirror (Fig. 5 ▶). Since this is the measured width it may be that not all the instrumental broadening contribution is utilized, most obviously that the contributing crystallites or crystallite cannot be distributed evenly over the full 35 µm. If we assume this full width at half-maximum of 0.023° comes from a single crystallite and take the average dimension 3.5 µm as a guide, then the instrumental broadening is reduced to 0.0115°, and the contribution of the crystallites or crystallite to the broadening is 0.0115°. This would equate to 0.7 µm-diameter spheres, if the contribution from the crystallites were from those satisfying the Bragg condition. Since, as discussed earlier, the absorption length in LaB_6_ is ∼1 µm and the maximum observable size would have to be less than this for the beam to get in and out, it is probably close to ∼0.7 µm. This explanation fits well to the assumption that these sharper peaks are from isolated crystallites that are close to the Bragg condition: subsequently confirmed by searching for these sharp peaks and observing the small peak shifts within the beam size. If the influence of absorption is ignored, and therefore the whole of the average crystallite width of 3.5 µm contributes 0.0026°, based on the Scherrer equation, and they are distributed over the 35 µm × 15 mm beam, then the peak width would be 0.021°, which by chance is close to that measured. It seems unlikely that so many similarly orientated crystals should exist within this area, and also this Scherrer width takes no account of absorption and extinction, both of which significantly increase this width. When more crystallites are included by rocking the sample then the profile width is close to 0.05°. If an arbitrary position on the sample is taken, for a stationary measurement, then the peak width can vary from 0.023 to 0.06° in 2θ. This suggests that the distribution of crystallites scattering close to but not exactly on the Bragg condition can change. This agrees with the observations discussed by Fewster & Andrew (1999 ▶).

Since it is possible to account for the scattering from a single crystallite, which fits that expected from this configuration, this greater width, ∼0.05°, is composed of an instrumental broadening contribution of 0.019° and crystallite diffraction broadening. Quite clearly these do not arise from contributions dominated by those close to the Bragg condition as this would only give rise to a peak width of <0.03°. This again suggests that the scattering is mainly from the intersection of the diffraction tails. Clearly with a very well defined beam, combined with the interpretation of the scattering process, it will be possible to extract detailed microstructure information.

### 5.4. Very rapid data collection

As indicated in the analysis of paracetamol, data can be collected in very short periods of time; the major peaks appear within a few seconds. This is exemplified by some rapid analyses of LaB_6_, which scatters more strongly. As discussed in the *Introduction* [Sec sec1], obtaining data at high resolution by conventional methods without scanning is not easy, because it is difficult to maintain the focusing condition without going to very large radii for both the source and the detector. Generally, without the focusing condition, the beam size at the detector is large and the resolution is poor. With the configuration presented in this article, this is not a restriction and, as illustrated already, data can be collected in parallel. This could have significant advantages for monitoring phase changes and other dynamic processes.

**Figure 13:**
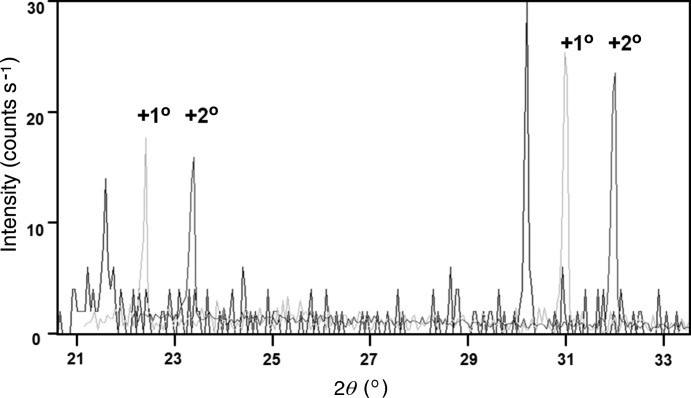
Data collected from LaB_6_ for various time frames; 0.5 s (no offset), 3 s (grey line offset +1°), 12 s (offset +2°). This indicates the possibilities in studying dynamic processes. The sample was rocked and the detector was stationary at a distance of 55 mm from the sample.

**Figure 14:**
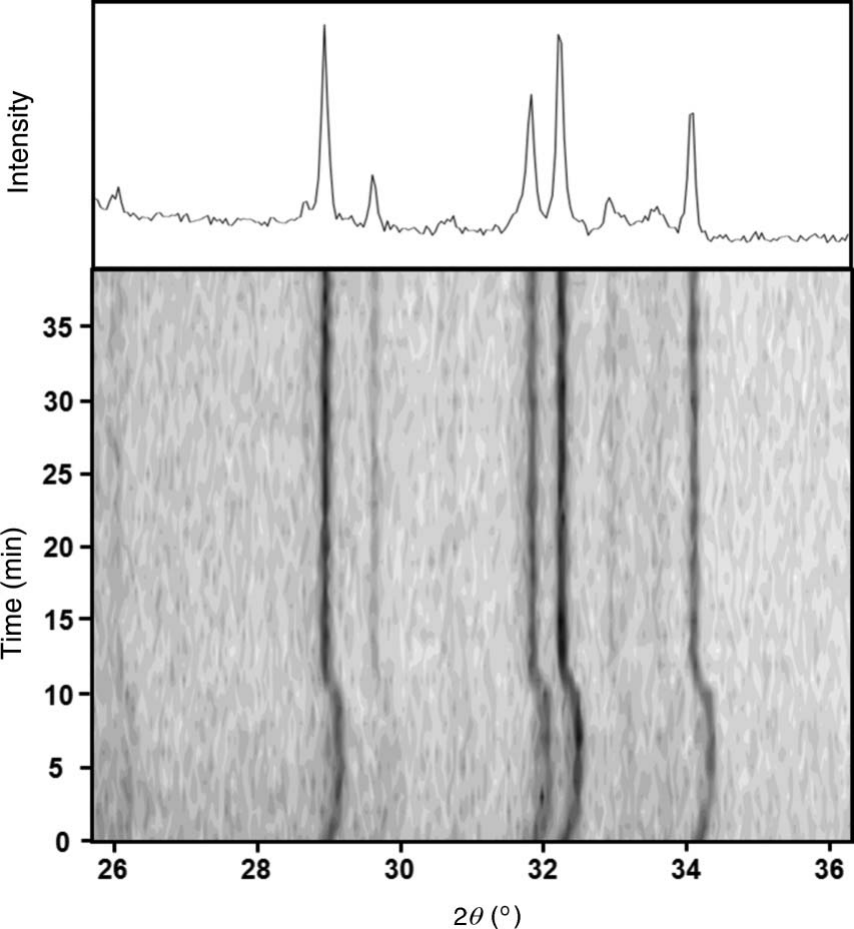
Data from cement after being sprayed with water, taken at 1 min intervals. This gives an indication of the change in lattice parameters during hydration and recovery after dehydration. The upper profile is the projection of the intensity from 15–40 min after dehydration. The sample was rocked during data collection and the start of data collection was ∼10 s after spraying.

A series of profiles have been collected for LaB_6_, for capture times of 0.5, 3 and 12 s to illustrate how quickly information can be obtained using a conventional laboratory sealed tube (Fig. 13 ▶). It is clear that the major contributions to the profile emerge from the background in less than a second for LaB_6_. Another example, given in Fig. 14 ▶, illustrates the scattering from cement after adding water. Cement powder was captured on adhesive tape, which was then sprayed with water, and data were measured at 60 s intervals. The influence of hydration and dehydration is observed in the 2θ range from 25.7 to 36.3°. The data were collected at a radius of 75 mm, giving a peak width of 0.14° for the 28.94° peak as a dry powder and 0.12° after this hydration and dehydration stage. Under certain dampening conditions this peak has been observed to have a full width at half-maximum of 0.065°, suggesting that the microstructure is dependent on these conditions. The sample was rocked throughout the data collection.

## 6. Conclusions

This paper presents a new high-resolution powder diffractometer that requires very simple sample preparation and mounting, and no sample alignment. It is very compact and collects data very rapidly. The basic principle with this method compared with other approaches is that the high resolution is achieved by creating a very small and very intense beam. This defines the resolution regardless of the lateral dimensions of the sample, so the sample can be large, and numerous crystallites can be brought into this beam by rocking to make the intensities reliable.

Interpretation of the scattering profiles relies on the idea that the scattering cannot come solely from scattering within the FWHM of the Bragg condition (P. Fewster, in preparation). By recognizing that the contribution is predominately from the tails of the diffraction profiles, data can be collected with relatively few crystallites and all the expected peaks appear so that ‘crystal statistics’ is not a serious issue. That the configuration without the mirror relies on scattering from 2000 crystallites further supports this thought.

Because the method does not depend on focusing, the resolution can be taken to the limit of 0.01° for this configuration by increasing the sample-to-detector distance, reducing the beam size onto the monochromator with slits or removing the mirror. The other significant advantage, because the method is independent of any focusing, is that the data can be collected in parallel with an array of detectors, making it possible to collect extremely high resolution data very quickly. The full pattern can be collected with and without the sample, allowing the removal of all the artefacts from the profile for a more complete analysis. This instrument provides a method for obtaining a full diffraction profile in a few seconds with a detector array. It could also facilitate continuous phase analysis, allowing levels of identification certainty to be monitored as data build up.

The likely errors associated with the method have been discussed, with an indication of how they can be compensated for by a full understanding and modelling of the whole instrument. The advantage of defining the scattering region in space to within 35 µm, as in these examples, is in eliminating sample alignment. However, if this dimension is reduced further to very small values, coupled to a higher intensity source and a long sample-to-detector distance, this will give exceptionally high resolution data and, because the sample can be rocked, reliable intensities can be achieved.

## Figures and Tables

**Table 1:** Comparison of intensity and data collection times, for similar statistical reliability, for the compact diffractometer (75 mm radius) and other commonly used powder diffractometers Based on an LaB_6_ sample that is assumed to have full coverage for all the diffractometers except the compact, which assumes 38% coverage.

Diffractometer	Intensity ratio: compact diffractometer (calculated)	Intensity ratio: compact diffractometer (measured)	Speed ratio: compact diffractometer (sequential 10.75°)	Intensity ratio: compact diffractometer (multistrip 10.75°)	Speed ratio: compact diffractometer (multistrip 107.5°)
Reflection Bragg–Brentano(240 mm)	0.241	–	61	1.06	8.1
Reflection Bragg–Brentano†(320 mm graphite monochromator)	0.162	0.145 (2)	42	0.75	5.1
Reflection Bragg–Brentano†(240 mm Ge 111 monochromator)	0.070	0.042 (5)	22	0.44	2.5
Transmission Bragg–Brentano(240 mm, *K*α_1_, 350 µm capillary)	0.702	–	180	3.6	24
Transmission Bragg–Brentano (240 mm, *K*α_1_, 700 µm capillary)	0.350	–	90	1.8	12

† Based on a large axial detector aperture of 27 mm compared with 14 mm for the compact diffractometer.

## Footnotes

1 To benefit from scanning in this mode and to capture all the data points 256 times, the scan has to start below and finish above the scan range required. However, as the range is increased, this overlap is only required at the beginning and end of the full scan and the impact of the additional range becomes less.
